# Surface Modification of Heterojunction with Intrinsic Thin Layer Solar Cell Electrode with Organosilane

**DOI:** 10.3390/mi15111339

**Published:** 2024-10-31

**Authors:** Bing-Mau Chen, Chih-Hung Chen, Shang-Ping Ying

**Affiliations:** Department of Semiconductor and Electro-Optical Technology, Minghsin University of Science & Technology, 1, Xinxing Road, Xinfeng, Hsin-Chu 30401, Taiwan; bmchen@must.edu.tw (B.-M.C.); seanvsko@gmail.com (C.-H.C.)

**Keywords:** heterojunction with intrinsic thin layer (HIT), indium tin oxide (ITO), self-assembled monolayers (SAMs), 3-aminopropyltrimethoxysilane (APTMS)

## Abstract

Solar cell (SC) technologies, which are essential in the transition toward sustainable energy, utilize photovoltaic cells to convert solar energy into electricity. Of the available technologies, heterojunction with intrinsic thin-layer (HIT) solar cells offers high efficiency and reliability. The current study explored the enhancement of HIT solar cell performance through the use of 3-aminopropyltrimethoxysilane (APTMS) self-assembled monolayers (SAMs) on the surface of the cells’ indium tin oxide (ITO) layer. Photoluminescence mapping revealed greater brightness and photocurrent in the HIT sample treated with APTMS SAMs, with the results indicating more favorable optical and electrical properties. The application of APTMS SAMs led to higher open-circuit voltage, fill factor, maximum power output, and efficiency by passivating the ITO surface and achieving energy level alignment, thereby enhancing the charge carrier dynamics. These findings demonstrate the potential of APTMS SAMs to improve HIT solar cell efficiency and reliability.

## 1. Introduction

Solar cell (SC) technologies, which use photovoltaic cells to convert solar energy into electricity, are critical in the transition toward sustainable energy. These technologies are among the most crucial renewable energy sources, alongside hydroelectric, wind, and geothermal energy. Solar cells are typically made from light-absorbing materials, such as silicon, which is abundant, highly reliable, and inexpensive to maintain. The development of solar energy is being driven by the need to address global challenges related to population growth, economic and environmental pressures, and the demand for green energy solutions. Solar energy systems offer considerable advantages, including efficiency, noiseless operation, and the ability to function under various weather conditions. As the solar industry has evolved, its focus has become improving efficiency, scaling production, exploring new materials, and reducing electricity costs. Silicon-based technologies including single crystalline silicon (c-Si), polycrystalline SCs, amorphous silicon (a-Si), dye-synthesized SCs, multijunction SCs, perovskite SCs, organic SCs, quantum dot SCs, and graded bandgap multilayer SCs have been thoroughly studied [1,2,3,4]. Such technologies have consistently dominated the market because of their high performance and eco-friendliness, and solar cell technologies are considered to be critical to achieving a future of sustainable energy.

**Figure 1:**
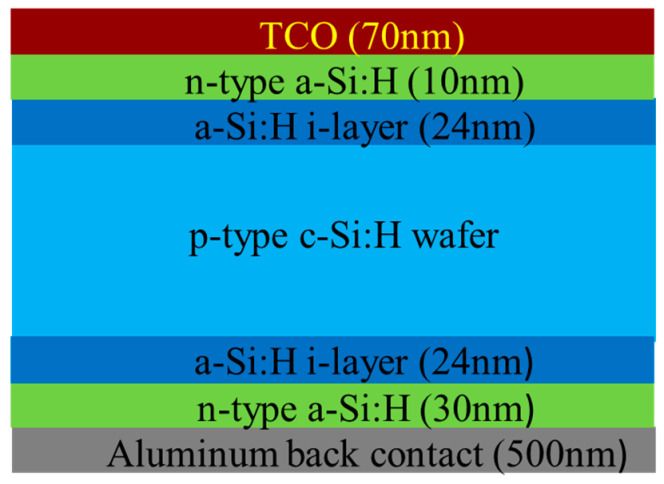
Schematic cross-sectional view of the heterojunction intrinsic thin layer solar cell in this study.

Heterojunction with Intrinsic Thin-layer (HIT) technology, which combines the beneficial properties of crystalline silicon with thin layers of amorphous silicon, represents a significant advancement in photovoltaic devices. HIT solar cells have achieved efficiencies exceeding 27% in laboratory conditions [5,6,7,8]. The structure of HIT cells typically includes a thin intrinsic layer of amorphous silicon placed between a crystalline silicon substrate and doped amorphous silicon layers, as depicted in Figure 1. This intrinsic layer acts as a passivation layer, reducing electron-hole recombination at the interface between different materials, improving the cell’s overall efficiency. Rather than relying on broader absorption through bandgap tuning, HIT cells primarily utilize the efficient photon absorption of crystalline silicon, while the amorphous silicon layers focus on enhancing surface passivation. Moreover, HIT cells exhibit excellent resistance to light-induced degradation (LID) and have a lower temperature coefficient than traditional silicon cells. The lower temperature coefficient helps reduce the negative effects of heat, ensuring that HIT cells maintain high efficiency even during prolonged sunlight exposure. Consequently, HIT cells offer superior long-term stability compared to traditional silicon-based solar cells.

Transparent conductive oxide (TCO) layers are essential components of HIT solar cells, playing a critical role in their performance and efficiency. TCO layers are materials that exhibit both high electrical conductivity and optical transparency, rendering them ideal for use as front and back contacts in photovoltaic devices. In HIT solar cells, TCO layers and a metal grid electrode are formed on the doped layers and are used as the front electrode; this setup enables the maximum amount of light to pass through to the active layers while simultaneously collecting and transporting generated charge carriers. The careful selection and optimization of TCO materials are crucial to achieving high efficiency and reliable performance in HIT photovoltaic devices. Common TCO materials include indium tin oxide (ITO), aluminum-doped zinc oxide, and fluorine-doped tin oxide. These materials are often selected for their excellent electrical properties, high transparency in the visible spectrum, and good chemical stability. ITO is currently the best choice for TCO electrode materials because of its high transmissibility, high conductivity, low light absorption, toughness, and high stability. ITO is used in various optoelectronic applications, such as electronic displays, heaters within window panes, architectural uses, automotive uses, sensors, flat panel displays, and solar cells [9,10,11]. However, a few critical problems, such as defects and thermal and chemical instability, restrict the widespread application of ITO layers [12].

Defects in ITO strongly affect its electrical, optical, chemical, and thermal properties. Oxygen vacancies and interstitial tin atoms within ITO can enhance its electrical conductivity by increasing the concentration of carriers, but excessive defects reduce mobility by acting as scattering centers. Optical transparency can be negatively affected by defect-induced localized states in the bandgap, and variation in defect concentration can influence the material’s refractive index. Additionally, defects reduce stability by serving as reaction sites, increasing the material’s susceptibility to corrosion and altering the reactivity of its surface. Defects scatter phonons, reducing conductivity and affecting heat dissipation, and they alter thermal expansion, potentially causing problems in thermal cycling. Common defects include oxygen vacancies, interstitial tin, grain boundaries, dislocations, and point defects, all of which must be carefully controlled if ITO is to be optimized for applications such as transparent conductive coatings, touch screens, and solar cells.

Several methods have been employed to modify the defects in ITO, with these methods involving doping control, precise control over parameters during deposition, and postdeposition treatments including oxygen plasma treatment [13,14,15,16,17]. Layer-by-layer growth methods provide favorable control over the thickness and uniformity of ITO films; such methods can modify the defects in ITO and enhance the material’s properties for specific applications [18]. However, technical and fabrication-related concerns act as a barrier to the full realization of commercial-grade photovoltaic devices. The most prominent challenge is that of achieving scalable and economically viable controlled fabrication at a low cost. Unlike the aforementioned techniques, chemical treatments such as hydrogenation and surface passivation can fill oxygen vacancies and reduce surface states, and these methods are suitable for large-scale production at a low cost. Self-assembled monolayers (SAMs) are an example of a chemical treatment technique. SAMs on ITO are primarily used to enhance surface properties to improve performance in electronic and optoelectronic applications by enhancing charge injection and transport efficiency in devices. They also facilitate surface modification by introducing functional groups that can enhance compatibility with and adhesion to other materials. SAMs protect ITO from environmental contaminants and oxidation, extending its lifespan and maintaining its electrical and optical properties. Furthermore, SAMs can optimize the interface between ITO and an organic active layer, improving charge collection efficiency and photovoltaic conversion efficiency. More importantly, SAMs can be deposited on ITO by immersing ITO-coated devices in SAM solution, an approach that is suitable for inexpensive large-scale production. Therefore, SAMs provide an effective and flexible method for improving ITO surface characteristics and device performance, particularly in high-performance optoelectronic devices.

Regarding the application of SAMs, the precise number of molecules that can be formed on ITO is continually increasing as researchers are developing new molecules and modifications for specific applications. Octadecylphosphonic acid, hexadecylphosphonic acid, and phenylphosphonic acid are generally used in SAMs to modify ITO, but 3-aminopropyltrimethoxysilane (APTMS) can also be considered. APTMS is an organosilane compound commonly used to modify surfaces to introduce amino functional groups. The structure of APTMS enables it to form strong covalent bonds with both inorganic surfaces and organic materials and thereby enhance the performance and functionality of devices. The amino groups in APTMS can enhance the adhesion of subsequently deposited layers, improving the quality of interfaces in multilayer structures. Moreover, amino-functionalized surfaces can improve charge injection and transport properties, making them suitable for use in optoelectronics. Therefore, APTMS is likely a favorable choice for fabricating SAMs on ITO. In this study, we investigated the optical and electrical properties of HIT devices containing SAMs on its ITO layer. APTMS was used as the material for the SAMs to modify the passivation of ITO on HIT devices.

## 2. Materials and Methods

**Figure 2:**
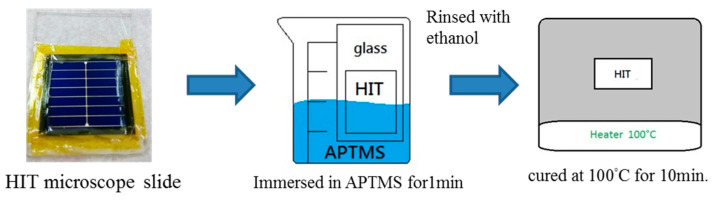
Schematic diagram of process flow charts in this study.

A schematic of the HIT structure in this study is presented in Figure 1. HIT solar cells of the size 2 × 2 cm^2^ were prepared on a p-c-Si wafer with a size of 4 inches and thickness of 165 µm. After initial wafer cleaning, intrinsic and doped amorphous silicon layers were deposited using plasma-enhanced chemical vapor deposition (PECVD). The substrates were placed in the loadlock chamber of the PECVD system under high vacuum. The sequential deposition of the intrinsic and n- a-Si layers was carried out using standard PECVD processes. The precursor gases, including semiconductor-grade silane (SiH_4_), phosphine (PH_3_), and diborane (B_2_H_6_), were diluted in hydrogen. Layers with varying thicknesses were deposited on the front and rear sides of the p-c-Si wafer. An ITO layer of 70 nm thickness was deposited through DC sputtering at a substrate temperature of 150 °C, which is used to improve film quality by enhancing crystallinity and conductivity. The mass ratio of the ITO target was In_2_O_3_:SnO_2_ = 90%:10%. Finally, the printed metal grid lines formed HIT solar cells. On the rear side of the wafer, an Al layer with a thickness of 500 nm was sputtered. The process of SAM fabrication on ITO by using APTMS comprised multiple steps, which are illustrated in Figure 2. The ITO-coated HIT sample was securely mounted on a microscope slide and thoroughly cleaned to eliminate any organic contaminants, ensuring a pristine surface for SAM formation. In the experiment, heat-resistant tape was used to carefully seal and fix the ITO-coated HIT sample above the microscope slide to prevent APTMS from seeping into the edge side of the ITO-coated HIT sample during the subsequent SAM formation process. The HIT sample coated with ITO was then immersed in 10% APTMS solution for 15 min, during which time APTMS molecules self-assembled into monolayers on the ITO surface. The ITO-coated HIT sample was subsequently rinsed with ethanol to remove any unbound APTMS molecules and dried under nitrogen flow. The ITO-coated HIT sample was cured at 100 °C for 10 min to ensure that the APTMS SAMs on the ITO surface were stable and well-ordered.

The cross-sectional morphology of the ITO-coated HIT sample was determined using scanning electron microscopy (SEM; JSM-6500F, JEOL-USA, Peabody, MA, USA). Water contact angle measurement is a simple yet powerful tool that can indicate the surface characteristics of ITO on HIT. In this study, such measurements were made using a protractor on a microphotograph. ITO-coated HIT samples with and without APTMS SAMs on the ITO surface were irradiated using a solar simulator (Oriel 91160A, Newport, Irvine, CA, USA; YSS-E40, Yamashita Denso Co., Ltd., Tokyo, Japan) under AM1.5G (100 mW/cm^2^) conditions. A Keithley 2400 was used to obtain current–voltage curves for the ITO-coated HIT samples with and without APTMS SAMs on their ITO. The dark current–voltage characteristic was also investigated to determine the electronic properties of ITO-coated HIT samples.

## 3. Results and Discussion

**Figure 3:**
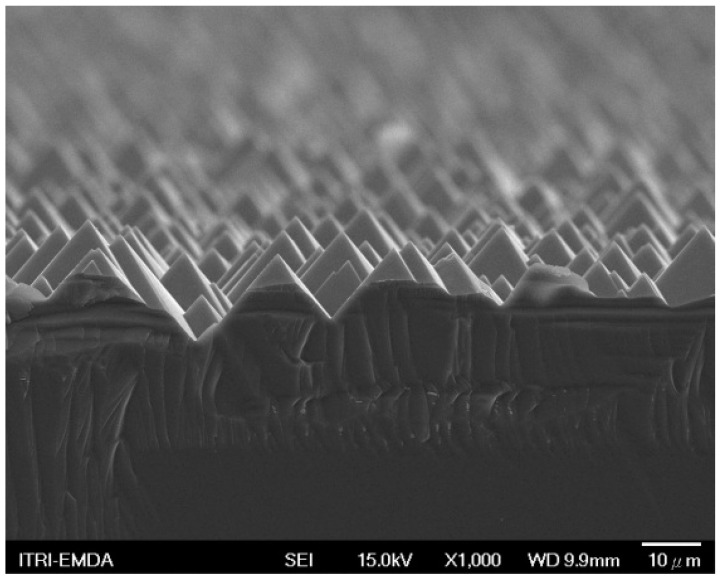
Scanning electron microscope cross-sectional image of APTMS SAMs on the ITO-coated HIT.

The cross-sectional SEM image of the APTMS SAMs on the ITO-coated HIT sample is presented in Figure 3. The SEM image shows a highly textured ITO surface with a pyramidal structure. This texture is characteristic of ITO layers in HIT solar cells, designed to increase surface area and reduce reflection, enhancing light absorption by the underlying photovoltaic material.

**Figure 4:**
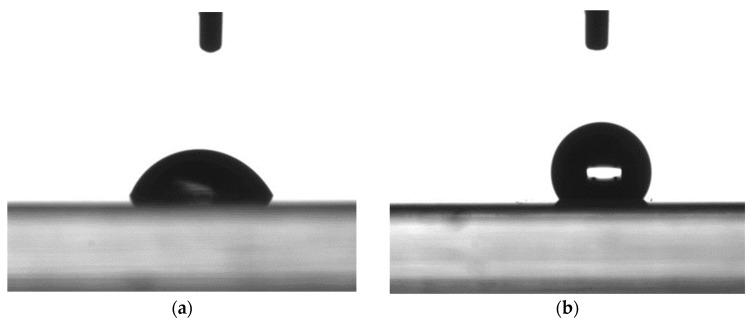
Microphotographs of water droplet on ITO-covered HIT solar cells (**a**) with and (**b**) without SAMs of APTMS on the ITO surface.

Water contact angle measurement is an essential tool for assessing the surface properties of materials, particularly their wettability and hydrophilicity. In this study, water contact angle measurement was employed to determine the hydrophilicity of the ITO-coated HIT solar cells and confirm the formation of APTMS SAMs on the ITO surface. Figure 4 presents microphotographs of a water droplet on ITO-coated HIT samples with and without APTMS SAMs. The water contact angle for the SAM-free ITO-coated HIT sample was measured as 127°, whereas that for the sample with APTMS SAMs was 66°. This much smaller contact angle indicated the greater hydrophilicity of the surface; the water droplet was more widely spread on the surface, suggesting a higher affinity between the surface and water molecules. The greater hydrophilicity of the ITO-coated HIT with APTMS SAMs was attributable to the surface chemistry introduced by the APTMS molecules. APTMS is an organosilane that contains nitrogen-containing functional groups such as amine (C-NH2), imine (C=NH), and sulfhydryl (C=N-OH), which are capable of forming hydrogen bonds with water molecules [19]. These functional groups increase the energy of a surface, making the surface more polar and thus more attractive to water. The finding of an improvement in hydrophilicity not only confirmed the success of APTMS SAM deposition on the ITO surface but also suggested potential benefits in terms of the performance of HIT solar cells. A more hydrophilic surface leads to stronger interactions between the ITO layer and subsequent layers in the solar cell structure, resulting in more favorable adhesion and potentially smaller losses due to interface-related recombination. These correspond to better charge transfer and higher collection efficiency, contributing to higher overall efficiency in the solar cell.

**Figure 5:**
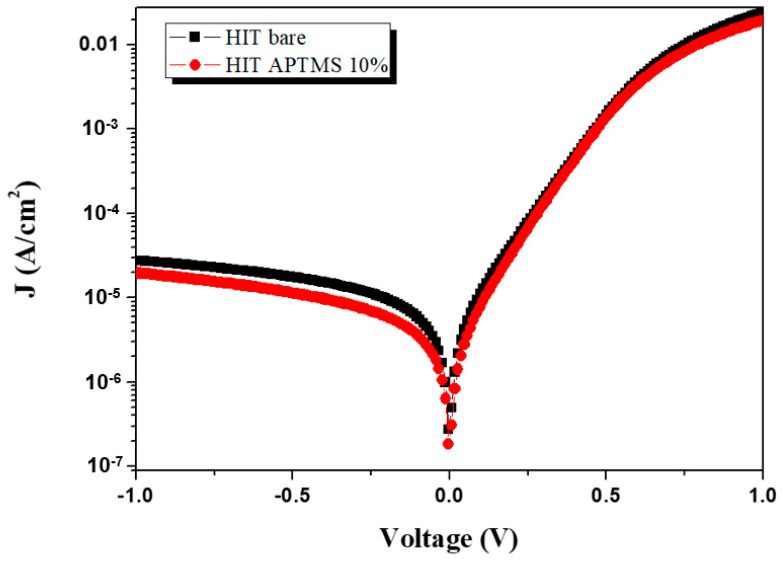
Dark current–voltage characteristics of the ITO-covered HIT solar cells with and without SAMs of APTMS on the ITO surface.

Figure 5 presents the dark I–V characteristics of the HIT solar cells in a semilogarithmic plot, where the applied voltage ranged from −1 to 1 V. The results are also summarized in Table 1. Through an examination of the dark I–V curve, this study was able to assess the extent of recombination losses and the effectiveness of charge carrier transport within the device. In a darkroom environment, the HIT solar cell with well-ordered APTMS SAMs on the ITO surface exhibited 29% lower dark current under reverse bias of −1.0 V than did the cell without APTMS SAMs. This considerably lower dark current indicated that the APTMS SAMs effectively reduced recombination losses of minority carriers [20,21,22]. In photovoltaic devices, dark current primarily arises from carrier recombination, a process in which electrons and holes combine without contributing to the photocurrent. A high recombination rate can result in poor device efficiency by decreasing the number of charge carriers available for power generation. The finding of a lower dark current under the condition of APTMS SAMs suggested that the monolayers provided effective surface passivation. Passivation involves minimizing the number of defect sites on a surface or at interfaces, where defects can act as recombination centers for charge carriers. In the current study, APTMS molecules attached to the ITO surface, filling its vacancies and reducing the number of trap states that would facilitate recombination. This improved surface quality resulted in fewer charge carriers lost to recombination, enhancing the overall efficiency of the solar cell. Additionally, the lower dark current when the APTMS SAMs were present indicated better charge carrier transport because fewer recombination events results in carriers being more likely to reach the electrodes. By reducing surface recombination, APTMS SAMs enhanced the open-circuit voltage and fill factor, leading to the more favorable performance of the HIT solar cell. Therefore, incorporating APTMS SAMs on the ITO-covered HIT solar cell effectively modifies the surface properties, leading to enhanced charge transport and less frequent recombination. This improvement in device performance underscores the potential of APTMS SAMs to optimize the efficiency of HIT solar cells.

**Figure 6:**
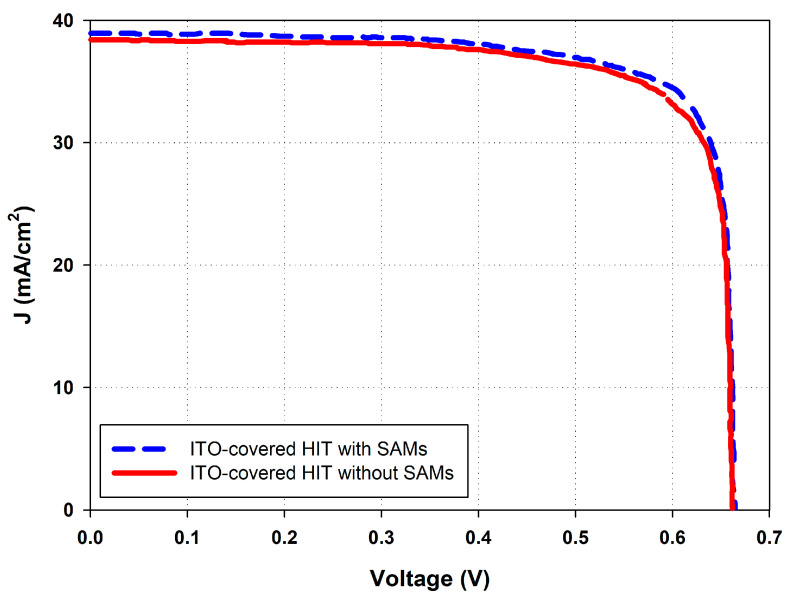
J−V characteristics of the ITO-covered HIT solar cells with and without SAMs of APTMS on the ITO surface.

**Figure 7:**
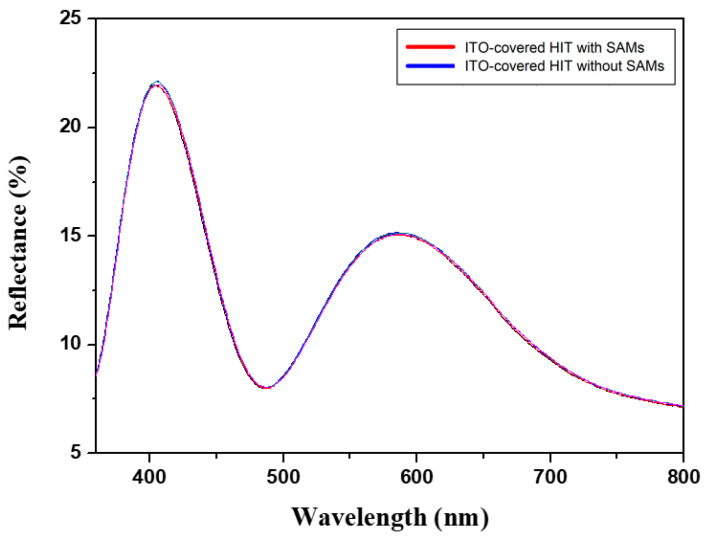
Reflectance of the ITO-covered HIT solar cells with and without SAMs of APTMS on the ITO surface.

Figure 6 presents the current–voltage characteristic curves of the ITO-covered HIT solar cells with and without APTMS SAMs on the ITO surface. The curves illustrate how the APTMS SAMs led to notable improvements in several key performance metrics of the solar cells. Table 2 summarizes the characteristic parameters derived from the I–V measurements, that is, the open-circuit voltage (Voc), fill factor (FF), maximum power output (P_MAX_), and overall efficiency (η). The solar cell treated with APTMS SAMs had more favorable values for all these parameters compared with those of the cell without APTMS SAMs. The higher Voc of the solar cell treated with APTMS SAMs indicated more favorable charge separation and smaller recombination losses; these results were obtained because the APTMS SAMs provided favorable surface passivation on the ITO, minimizing defect-induced carrier recombination. The cells also had better FF, suggesting more efficient charge carrier extraction and transport, likely due to more optimal energy level alignment being facilitated by the SAMs. The cells had superior P_MAX_ and η, which further corroborated the finding of overall cell performance enhancement because the APTMS SAMs contributed to more efficient light absorption and charge collection. The reflectance measurement of the ITO-covered HIT solar cells with and without APTMS SAMs on the ITO surface is presented in Figure 7. The small difference in reflectance suggests that applying APTMS SAMs slightly reduces reflection, which is expected to marginally enhance light absorption in the solar cell. In general, lower reflectance allows more light to be absorbed by the solar cell, potentially increasing Jsc. In the ITO-covered HIT solar cells, the slight reduction in reflectance with APTMS SAMs indicates that more light is entering the cell and being absorbed, which could result in a modest improvement in photocurrent and overall solar cell performance.

**Figure 8:**
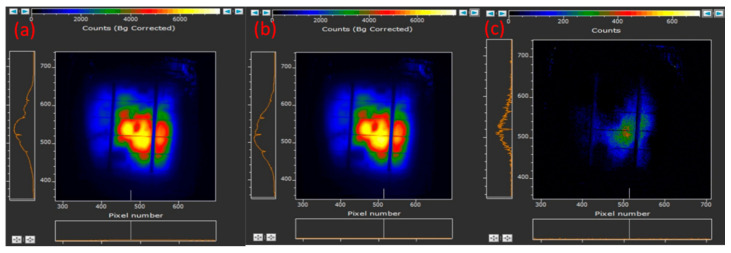
(**a**) PL mapping of HIT solar cells without the application of APTMS SAMs on the ITO surface, (**b**) PL mapping of HIT solar cells with the application of APTMS SAMs, (**c**) The subtraction of the PL mapping for (**a**) from (**b**).

Photoluminescence (PL) mapping has emerged as an invaluable tool for characterizing the ITO layer in HIT solar cells. This technique provides spatially resolved luminescence data, enabling researchers to detect variation in optical properties, identify defects, and assess the uniformity of an ITO surface [23]. PL mapping is particularly effective for examining the impact of surface treatments, such as the deposition of SAMs, on an ITO layer’s performance. A home-made photoluminescence (PL) measurement system using a laser diode with a wavelength of 808 nm and an output power of 50W (DILAS, Bozeman, MT, USA) as the excitation source, coupled with a 400-micrometer SMA fiber for precise light delivery onto the sample, was employed in this study. The emitted PL signal was captured by the highly sensitive CCD camera (iKon-M 934 series, Andor Technology Ltd., South Windsor, CT, USA). With the integration of sample temperature control (5300 series, Arroyo Instruments, San Luis Obispo, CA, USA) and analysis software, our system provides high-resolution, spatially resolved PL data, enabling detailed material and defect analysis in solar cells. Figure 8a,b presents the PL mapping measurement results obtained for HIT solar cells with and without APTMS SAMs on the ITO surface, measured under identical optical excitation power. These images provide a visual representation of the luminescent activity across the solar cell’s surface, offering insights into the distribution and intensity of light emission. The PL map of the HIT solar cells without APTMS SAMs indicated a baseline level of luminescence, reflecting the inherent optical properties and defect density of the untreated ITO layer [Figure 8a]. This baseline served as a reference for evaluating the effects of the SAMs. Figure 8b presents the map obtained for the cell with APTMS SAMs and indicates higher PL brightness across the HIT solar cell’s surface. This stronger luminescence was attributable to the improved optical properties resulting from the APTMS treatment. The SAMs passivated surface defects on the ITO, reducing the number of nonradiative recombination sites that would typically result in the quenching of luminescence. Consequently, radiative recombination was more frequent, leading to brighter PL emission. Figure 8c illustrates the subtraction of the PL mapping data for no APTMS deposition from the data for APTMS deposition and highlights the regions in which the luminescence was stronger due to SAM treatment, clearly demonstrating the beneficial impact of the APTMS SAMs on the ITO surface. The stronger PL intensity was indicative of improved charge carrier dynamics because fewer surface defects allowed for better charge carrier collection and transport. The higher PL brightness not only signified an improvement in the optical properties but also correlated with the higher electrical performance of the HIT solar cell. By passivating defects and optimizing surface properties, APTMS SAMs contribute to higher photocurrent generation and efficiency. This illustrates the dual role of APTMS—to enhance both optical and electrical properties. PL mapping is a powerful tool for evaluating and optimizing surface modifications in photovoltaic devices.

## 4. Conclusions

This study demonstrated that applying APTMS SAMs to the ITO surfaces in HIT solar cells considerably improves the optical and electrical properties of these cells. The PL mapping results provide valuable insights into how APTMS passivates surface defects and optimizes energy level alignment, leading to more favorable open-circuit voltage, FF, maximum power output, and efficiency. The findings suggest that APTMS SAMs can effectively enhance charge carrier collection and transport, contributing to the development of more efficient and reliable photovoltaic devices. By providing a cost-effective and scalable method for improving the properties of ITO surfaces, APTMS SAMs hold promise for advancing HIT technology and facilitating the broader adoption of sustainable energy solutions.

## Figures and Tables

**Table 1:** Dark current-voltage characteristics of the ITO-covered HIT solar cells with and without SAMs of APTMS on the ITO surface.

	J (μA/cm^2^)				
	−1.0 V	−0.8 V	−0.6 V	−0.4 V	−0.2 V
ITO-covered HIT without SAMs of APTMS	−27.6	−24.1	−19.9	−15.4	−9.81
ITO-covered HIT with SAMs of APTMS 10% for 10 min	−19.6	−16.5	−13.2	−9.79	−6.02
ΔJ (%)	29	31.5	33.6	36.4	38.6

**Table 2:** J−V characteristics of the ITO-covered HIT solar cells with and without SAMs of APTMS on the ITO surface.

	V_oc_ (mV)	J_sc_ (mA/cm^2^)	F.F. (%)	P_Max_ (mW)	Efficiency (%)	R_P_ (Ω)	R_S_ (Ω)
ITO-covered HIT without SAMs of APTMS	660	38.5	78.7	80.2	20.1	160	0.00464
ITO-covered HIT with SAMs of APTMS 10% for 10 min	662	38.9	80.4	82.5	20.6	247	0.00296

## Data Availability

The data presented in this study are available on request from the corresponding author.
